# Cost-effectiveness and cost-utility analysis of OTC use of simvastatin 10 mg for the primary prevention of myocardial infarction in Iranian men

**DOI:** 10.1186/s40199-015-0129-2

**Published:** 2015-12-30

**Authors:** Mohammadreza Amirsadri, Abbas Hassani

**Affiliations:** Department of Clinical Pharmacy and Pharmacy Practice, Faculty of Pharmacy and Pharmaceutical Sciences, Isfahan University of Medical Sciences, Isfahan, Iran

**Keywords:** Cost-effectiveness, Cost-utility, myocardial infarction, Markov model, Primary prevention, Simvastatin, Over-the-counter

## Abstract

**Background:**

Several clinical trials and meta-analyses have shown the advantageous effects of statins in populations with different levels of cardiovascular disease (CVD) risk. Considering the increasing cardiovascular risk among the Iranian population, the cost-effectiveness of the use of simvastatin 10 mg, as an Over-The-Counter (OTC) drug, for the primary prevention of myocardial infarction (MI) was evaluated in this modeling study, from the payer's perspective. The target population is a hypothetical cohort of 45-year CVD healthy men with an average (15 %) 10-year CVD risk.

**Methods:**

A semi-Markov model with a life-long time horizon was developed to evaluate the Cost-Utility-Analysis (CUA) and Cost-Effectiveness-Analysis (CEA) of the use of OTC simvastatin 10 mg compared to no-drug therapy. Two measures of benefits were used in the model; Quality-Adjusted-Life-Years (QALYs) for the CUA and Life-Years-Gained (LYG) for the CEA. To examine the robustness of the results, one-way sensitivity analysis and probabilistic sensitivity analysis were applied to the model.

**Results:**

For the base-case scenario with a discount rate of 0 % the estimated ICERs were 1113 USD/QALY and 935USD/LYG per patient (using governmental tariffs).

No threshold has been determined in Iran for the cost-effectiveness of health-related interventions. However, according to the recommendation of WHO, this intervention can be considered highly cost-effective as its ICER is far less than the reported GDP per capita for Iran by World bank in 2013 ($4763).

**Conclusions:**

This modeling study showed that the use of an OTC low dose statin (simvastatin 10 mg) for the primary prevention of myocardial infarction (MI) in 45-year men with a 10-year CVD risk of 15 % could be considered highly cost-effective in Iran, as it meets the WHO threshold of the annual GDP per capita ($4763).

## Background

Chronic non-communicable diseases (NCDs) are universally recognized as the major causes of death and disability [1]. In 2008 around 48 % of NCD-related deaths were reported to be due to cardiovascular diseases (CVDs). It is predicted that by 2020, CVDs will be responsible for three-quarters of all deaths in countries with low- and middle-income [2].

Cardiovascular diseases are the most preventable causes of death in both developed and developing countries. The majority of CVD conditions with modifiable risk factors such as hypertension, dyslipidemia, obesity and diabetes are preventable or controllable [3]. Several clinical trials and meta-analyses have shown the advantageous effects of statins for the primary prevention of CVDs among populations with different levels of CVD risk [4]. Statin**s** are a class of pharmaceuticals used to lower LDL-cholesterol levels by inhibiting of the enzyme HMG-COA reductase [5]. Statins, independent of their lipid-lowering effect, may also improve endothelial function, inhibit inflammatory responses, stabilize atherosclerotic plaques and show vasculo-protective actions [6]. The indication for the use of statins also have been suggested for patients with even low normal LDL cholesterol levels in hopes of favorably altering the incidence of CVDs [7, 8].

To decrease the risk of a first major CVD event in people who are at moderate risk, the UK medicines and healthcare products regulatory agency reclassified simvastatin 10 mg (Zocor Heart-Pro) as an over the counter(OTC) medicine in 2004. The target population includes men aged 55 or more, men aged 45 to 54 years with one or more risk factor, and women aged 55 or more with one or more risk factor [9].

With respect to the increasing prevalence of CVDs among the Iranian population and its accrued costs, the cost-effectiveness and cost-utility of the use of 10 mg simvastatin among 45-year Iranian men with an average (15 %) 10-year CVD risk from the perspective of payer were estimated in this study [10].

## Methods

The population of this study includes a hypothetical cohort of CVD-healthy men aged 45 with a 10-year CVD risk of 15 %.

For chronic disease with recurrent events like as CVD, particularly when the risk of the disease progression persists indefinitely, Markov modeling is generally the preferred choice. Markov models with the transition probabilities which change with respect to time are named semi-Markov models [11].

A semi-Markov model was developed to evaluate the cost-effectiveness and cost-utility of the use of OTC simvastatin10mg (a low dose statin) for the primary prevention of myocardial infarction (MI) compared to no drug-therapy.

The main measured consequences were LYG for the CEA and QALY for the CUA.

Life-years-gained is a measure of the benefits from use of an intervention in terms of increased average life expectancy or delay of death in the population when compared with the alternative intervention [12].

Quality-adjusted-life-year is used to illustrate the outcomes of health care programs through adjusting the life years gained by an estimate of utility generally measured using a preference based method [13]. QALYs gained with treatment therefore incorporate benefits in both quantity and quality of life.

**Fig. 1:**
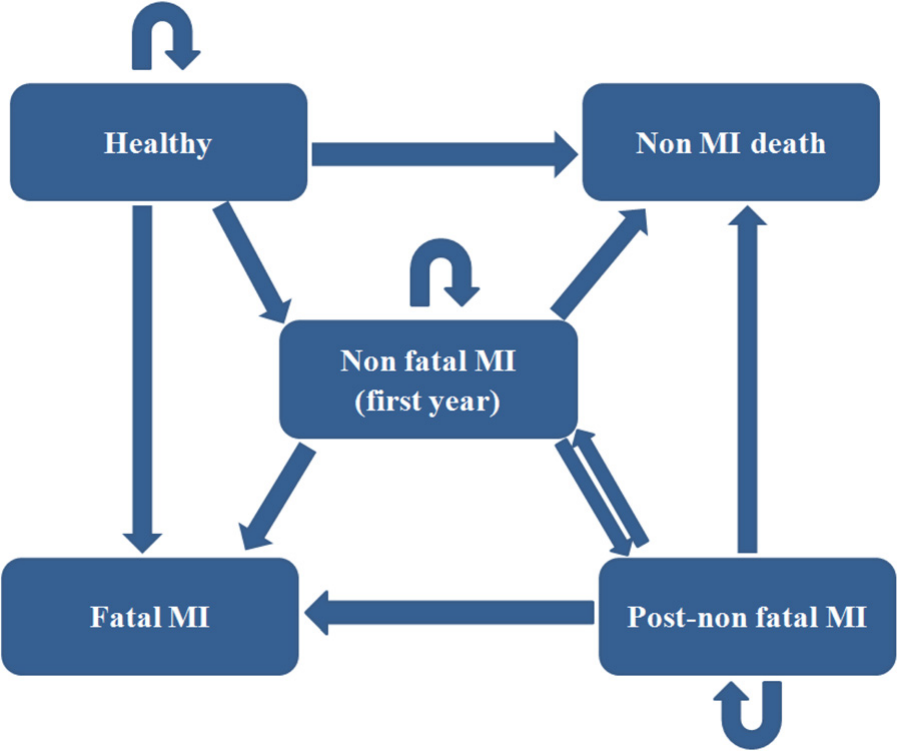
The Markov model diagram

The choice of cycle length depends upon the interventions of interest as well as the type of disease [14]. For models with life-long time horizon and relatively rare events the cycle length can be one year [15]. Majority of modeling studies on CVD adopted a cycle length of one year [16]. The current model assumes Markov cycles of one year and consists of 5 different health states including: healthy, non-fatal MI (first year), post-MI, fatal-MI and death due to any reason other than MI (to prevent double counting). As in the following years after an acute (first year) MI, both the treatment costs and the probability of a recurrent MI are different from the first year; separated health states for this were considered in the model (Fig. 1).

In this model, each individual starts as a CVD-healthy person. A healthy person might develop a non-fatal MI, die from a fatal MI, or die for any reason other than MI. Otherwise he would be transferred to the next cycle as a healthy person. If a patient develops a non-fatal MI, the patient might experience a new non-fatal MI, die due to a fatal MI or die for other reasons. If none of these happened, the patient would be transferred to the next cycle with a history of MI (post-MI) with the probabilities and costs related to this health state (which are different from the first-year MI). Possible transitions from post-MI to other health states are similar to those of non-fatal MI.

Once patients experience an MI event, they would receive a POM (Prescription-Only-Medicine) statin (atorvastatin 10 mg) in both intervention and no-intervention groups for life time.

The time horizon of an economic evaluation should be long enough to be able to capture both the major costs and the major future outcomes of treatment including the benefits, potential side effects, morbidity and mortality. Therefore in many cases a patient’s life time is the preferred time horizon for the study [14, 17].

Like as many economic evaluation studies on CVD, this model would be continued until 100 year of age (when most of the cohort have died) or death [16]. However, due to the nature of Markov models, some proportion of the cohort remain alive, regardless of how high the applied mortality rates are [18].

For the base-case, a moderate 10-year total CVD risk of 15 % was taken into account. As the CVD risk rises with age, an annual increase of 0.03 % in CVD risk was considered in the model [16]. The proportions of fatal and non-fatal MI events among total CVD events were sourced from the Isfahan Cohort Study (ICS) [19]. Different scenarios were evaluated in this modeling study. For consistency with national studies, a second scenario was evaluated in which the probabilities of fatal and non-fatal MI, independent of base-line CVD risk, were sourced from the ICS population.

As people aged 70 or more could be considered at high risk of CVD, in a third scenario, a POM statin (atorvastatin 10 mg) was prescribed from 70 years of age for the primary prevention, in both intervention and no-intervention groups [20].

Three different scenarios of discounting were also considered in this study including: no discounting (0 %), a discount rate of 3 % for both costs and effects (following recommendations of the WHO-CHOICE) [21] and a discount rate of 7.2 % for costs and 3 % for effects, according to a domestic study [22].

Input parameters, including transition probabilities, relative risks related to treatment with statins and the related sources of data are illustrated in Tables 1 and 2.

**Table 1:** Relative risks for the use of statins

The RR^a^ for the use of simvastatin 10 mg	
healthy to non-fatal MI	0.752
healthy to fatal MI	0.813
The RR^b^ for the use of atorvastatin 10 mg	
healthy to non-fatal MI	0.656
healthy to fatal MI	0.740

^a^RR = Relative risk, ^b^data sourced from reference 16

**Table 2:** The transition probabilities applied in the model

TP/age	45-49	50-54	55-59	60-64	65-69	70-74	75-79	80-84	85-100	Reference
MI To MI	0.1280	0.1280	0.1152	0.1152	0.1019	0.1019	0.0874	0.0874	0.0711	[16]
MI To FMI	0.0224	0.0348	0.0348	0.0700	0.0700	0.1054	0.1054	0.1270	0.1270	[34–37]
Post-MI To MI	0.0162	0.0162	0.0179	0.0179	0.0185	0.0185	0.0178	0.0178	0.0160	[16]
Post-MI To FMI	0.0052	0.0052	0.0092	0.0092	0.0152	0.0152	0.0235	0.0235	0.0340	[16, 38]
Non MI death	0.0028	0.0043	0.0056	0.0084	0.0131	0.0213	0.0426	0.0705	0.1143	[39, 40]
Healthy To MI (ICS)	0.0031	0.0031	0.0044	0.0044	0.0094	0.0094	0.0061	0.0061	0.0061	[19]
Healthy To FMI (ICS)	0.0015	0.0015	0.0050	0.0050	0.0082	0.0082	0.0080	0.0080	0.0080	[19]

*TP* = transition probability, *MI* = non-fatal myocardial infarction in first year, *FMI* = fatal myocardial infarction, *Post-MI* = subsequent years of non-fatal myocardial infarction, *ICS* = Isfahan Cohort Study

To calculate QALYs, a utility weights of 0.76 for MI [16] and 0.88 for post-MI [23] were applied to the model. To account for diminishing in health with age, age-related utility weights were also applied to the model. These utility weights were taken from the Ward et al. study on statins in 2007 [16].

This study was conducted from the payer's perspective. Direct costs including drug acquisition costs, laboratory tests, para-clinical examinations, physician's visits and hospitalization costs were taken into account. Considering the selected perspective, indirect costs were not investigated in this study. The costs were expressed in USD, considering an exchange rate of 26,912 Iranian Rials for each USD. The applied exchange rate was the monthly average (from 22.11.2014 to 22.12.2014) reported by the central bank of Iran [24].

To estimate the unit costs of treatment, we sought expert clinical advice from the cardiologists of Isfahan University of Medical Sciences teaching hospitals (Alzahra hospital and Chamran hospital). The treatment tariffs were sourced from the last published tariff books by the Iranian Ministry of Health and Medical Education. Also, in different scenarios, two separated series of tariffs for private and governmental sections were taken into account [25]. The acquisition cost of each OTC simvastatin 10 mg tablet (1,100 Rials = 0.041 USD) sourced from the Food and Drug Organization [26].

For those who received POM statin (atorvastatin 10 mg) for the primary prevention from 70 years of age, 4 annual general practitioner visits and two sets of liver function enzyme tests (SGOT and SGPT) were taken into account.

Table 3 shows the treatment costs used in the model.

**Table 3:** The treatment costs for the first year and following years of MI. The costs in this table were obtained from references 32 & 33

	Governmental					Private				
		MI (first year)		Post- MI			MI (first year)		Post-MI	
	Cost ($)	Number of cost units	Total costs ($)	Number of cost units	Total costs ($)	Cost ($)	Number of cost units	Total costs ($)	Number of cost units	Total costs ($)
CCU hospitalization fee (per day)	77.59	2	155.17	-	-	230.75	2	461.50	-	-
General care units hospitalization fee (per day)	60.87	2	121.73	-	-	180.59	2	361.18	-	-
Consultant visit fee	3.72	7	26.01	2	7.43	9.66	7	67.63	2	19.32
General practitioner visit fee	2.97	3	8.92	4	11.89	6.13	3	18.39	4	24.52
-Para-clinical examinations:										
Electrocardiogram	3.27	9	29.43	2	6.54	7.43	9	66.88	2	14.86
Echocardiography	35.97	1	35.97	-	-	81.75	1	81.75	-	-
Exercise tolerance test	18.64	1	18.64	-	-	42.36	1	42.36	-	-
-Medical laboratory tests:										
Lab. patient admission fee	0.45	3	1.34	2	0.89	0.97	3	2.90	2	1.93
Lab. service fee	0.00	3	0.00	2	0.00	0.74	3	2.23	2	1.49
CBC Dif.	0.74	3	2.23	2	1.49	2.01	3	6.02	2	4.01
BUN	0.41	3	1.23	2	0.82	0.89	3	2.68	2	1.78
Cr	0.52	3	1.56	2	1.04	1.08	3	3.23	2	2.16
Na	0.59	3	1.78	2	1.19	1.30	3	3.90	2	2.60
K	0.59	3	1.78	2	1.19	1.30	3	3.90	2	2.60
BS	0.45	3	1.34	2	0.89	0.97	3	2.90	2	1.93
TG	0.71	3	2.12	2	1.41	1.56	3	4.68	2	3.12
Cholesterol	0.52	3	1.56	2	1.04	1.11	3	3.34	2	2.23
PT INR	0.93	1	0.93	-	-	1.82	1	1.82		
PTT	0.93	1	0.93	-	-	1.82	1	1.82	-	-
Troponin	2.45	2	4.90	-	-	8.62	2	17.24	-	-
LDH	1.86	1	1.86	-	-	4.35	1	4.35	-	-
CPK	2.49	1	2.49	-	-	5.39	1	5.39	-	-
SGOT	0.63	3	1.90	2	1.26	1.45	3	4.35	2	2.90
SGPT	0.63	3	1.90	2	1.26	1.45	3	4.35	2	2.90
ESR	0.26	1	0.26	-	-	0.56	1	0.56	-	-
-Pharmaceuticals:										
ASA 80	0.01	365	3.66	365	3.66	0.01	365	3.66	365	3.66
Clopidogrel	0.29	365	105.79	-	-	0.29	365	105.79	-	-
Metoprolol	0.01	365	4.61	365	4.61	0.01	365	4.61	365	4.61
Enoxaparin	3.72	1	3.72	-	-	3.72	1	3.72	-	-
Atorvastatin10	0.03	365	11.94	365	11.94	0.03	365	11.94	365	11.94
Ranitidine	0.02	30	0.60	-	-	0.02	30	0.60	-	-
Oxazepam	0.01	4	0.04	-	-	0.01	4	0.04	-	-
Captopril	0.01	4	0.05	-	-	0.01	4	0.05	-	-
Streptokinase	9.29	1	9.29	-	-	9.29	1	9.29	-	-
Drug dispensing fee	0.20	6	1.18	6	1.18	0.59	6	3.57	6	3.57
Total			566.85		59.74			1318.62		112.14

We examined the effect of changing several different parameters in one-way (univariate) sensitivity analyses, for the base-case scenario. Results from the one-way sensitivity analyses are presented as Tornado charts.

Also parameter uncertainty was dealt with by probabilistic sensitivity analysis for the base-case scenario, using Monte Carlo simulation with 10,000 iterations for each evaluation. For each iteration, a value of each input variable was selected randomly from its distribution (lognormal distribution for relative risks and costs and beta distribution for transition probabilities) [27]. The parameters that we varied in the probabilistic sensitivity analysis (PSA) included relative risks of the use of statins for myocardial infarction (±10 %), secondary MI transition probabilities (±10 %), OTC statin tablet cost (±25 %), total MI and post-MI treatment costs (±20 %). Results from the PSA are presented as scatter plots of incremental cost-effectiveness ratios for QALY and LYG.

## Results

Different scenarios were evaluated in this study including the base-case, ICS scenario, in which the primary transition probabilities were sourced from the ICS study, and a scenario in which patients in both groups received a POM statin for the primary prevention from 70 years of age. Also three different discount rates and two types of tariffs (governmental and private) were examined for each scenario. Table 4 shows the obtained results for these scenarios.

**Table 4:** Final results of different scenarios

		Cost (USD/Patient)				Effect (Per Patient)				Incremental results (Per Patient)			
		Governmental tariffs		Private tariffs		QALY		LYG		Inc-Cost/QALY		Inc-Cost/LYG	
Discount rate	Scenario	No-drug therapy	OTC statin	No-drug therapy	OTC statin	No-drug therapy	OTC statin	No-drug therapy	OTC statin	Governmental tariffs	Private tariffs	Governmental tariffs	Private tariffs
0 %	Base-case	214.70	652.05	445.21	830.02	26.37	26.77	33.82	34.29	1113.40	979.65	935.12	822.79
	ICS	261.18	676.05	543.45	896.28	25.84	26.31	33.13	33.69	884.99	752.65	736.79	626.62
	POM statin from 70	463.59	768.23	835.25	1107.22	26.50	26.80	33.98	34.34	1001.43	894.04	863.83	771.19
3 %	Base-case	110.71	384.95	231.85	477.97	16.56	16.74	20.71	20.91	1567.74	1407.00	1374.03	1233.16
	ICS	128.68	394.62	270.32	504.34	16.37	16.57	20.46	20.70	1309.20	1152.07	1131.89	996.04
	POM statin from 70	204.12	428.42	378.37	581.72	16.60	16.75	20.76	20.93	1526.75	1384.16	1369.28	1241.40
7.2 % for costs & 3 % for effects	Base-case	54.91	227.23	116.64	274.35	16.56	16.74	20.71	20.91	985.12	901.62	863.40	790.21
	ICS	59.31	229.65	126.25	281.06	16.37	16.57	20.46	20.70	838.56	762.14	724.99	658.92
	POM statin from 70	81.14	239.40	157.83	303.41	16.60	16.75	20.76	20.93	1077.21	990.87	966.11	888.68

**Fig. 2:**
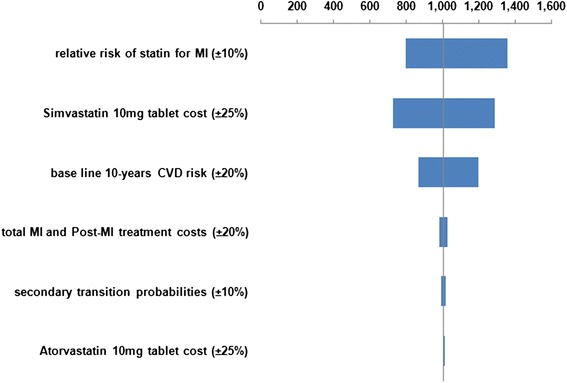
Tornado chart for Incremental cost/QALY per patient (governmental tariffs)

**Fig. 3:**
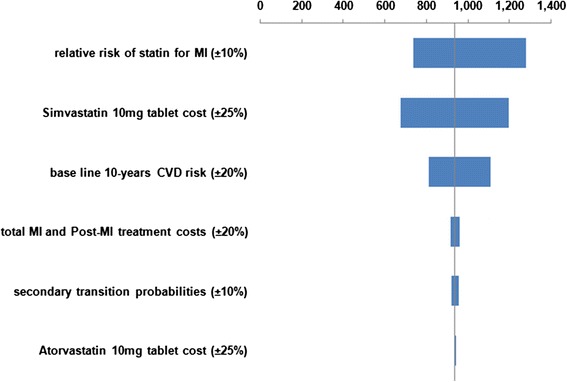
Tornado chart for Incremental cost/LYG per patient (governmental tariffs)

**Fig. 4:**
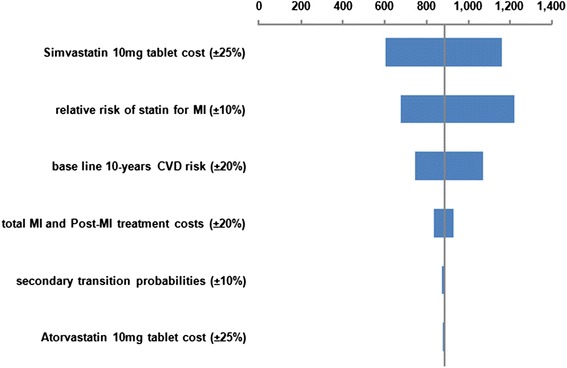
Tornado chart for Incremental cost/QALY per patient (private tariffs)

**Fig. 5:**
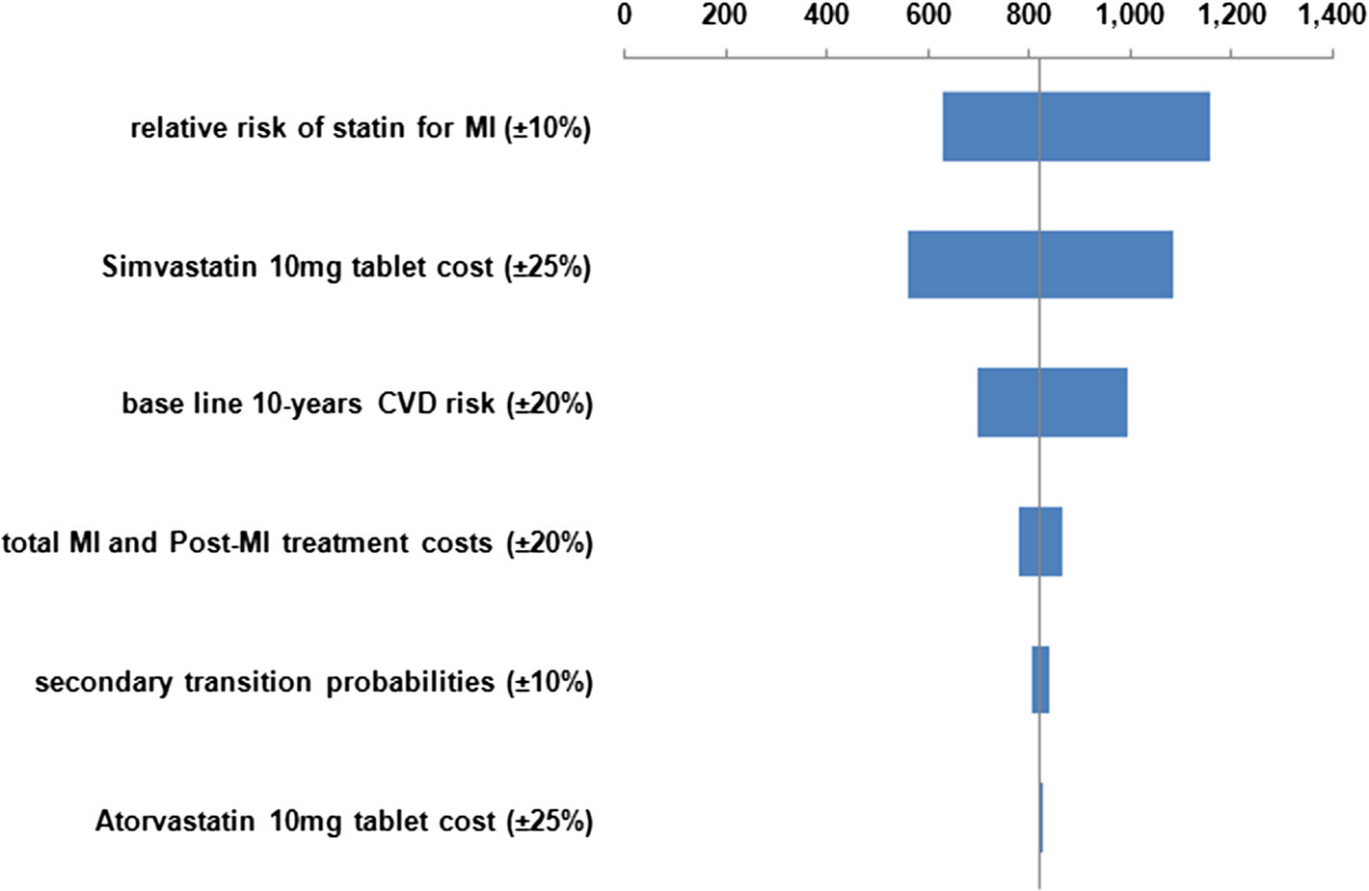
Tornado chart for Incremental cost/LYG per patient (private tariffs)

Figures 2, 3, 4 and 5 illustrate the Tornado charts for the one-way sensitivity analyses. The evaluated data and the examined range for each of them are shown in the charts. The Tornado charts show that the incremental-cost-effectiveness-ratios (ICERs) most affected by the relative risk of the use of statin and the cost of the OTC statin tablets.

**Fig. 6:**
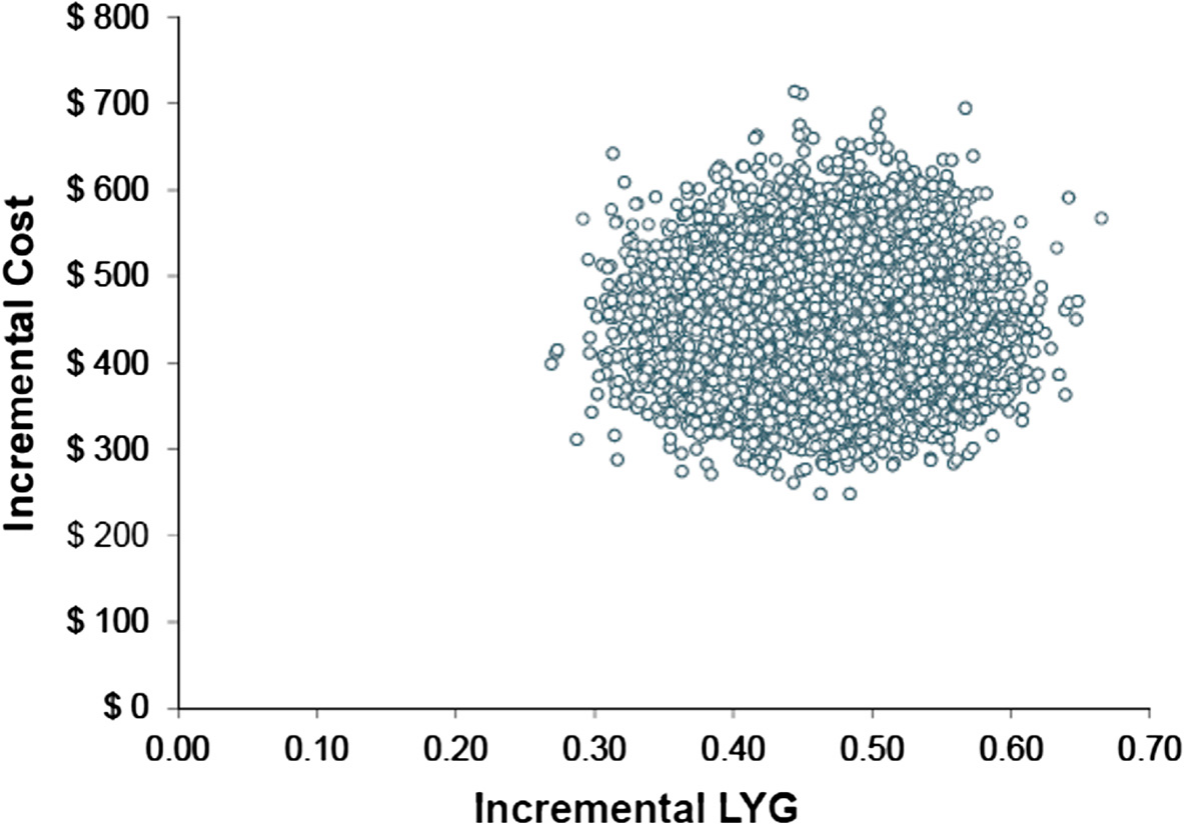
PSA Scatter plot of Incremental cost/LYG ratio (governmental tariffs)

**Fig. 7:**
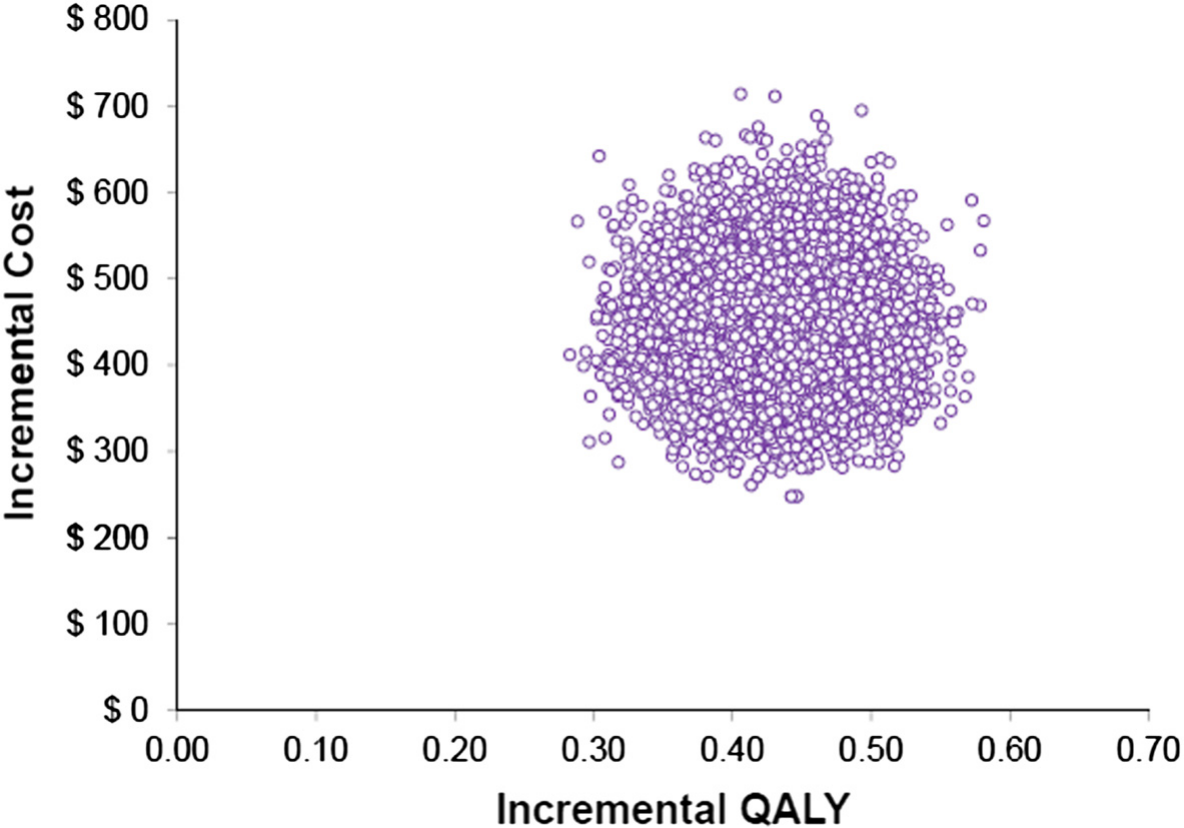
PSA Scatter plot of Incremental cost/QALY ratio (governmental tariffs)

Figures 6 and 7 for the probabilistic sensitivity analysis show that the use of OTC simvastatin 10 mg, compared with no-drug therapy for the primary prevention in 45-year men with a CVD 10-risk of 15 %, resulted in higher costs and more LYG and QALYs gained in all of the simulations. According to the probabilistic sensitivity analysis, estimated incremental cost per QALY gained and incremental cost per LYG are $1138 (95 % confidence interval [CI]: $797-$1595) and $960 (95 % confidence interval [CI]: $663-$1363), respectively. In addition, all the points comparing OTC simvastatin 10 mg with no-drug therapy for the primary prevention fell below the recommended threshold of WHO of GDP per capita (the reported GDP per capita by World bank for Iran in 2013 ($4763)) which means the intervention is highly cost-effective [28].

A cost-effectiveness acceptability curve (CEAC) illustrates the probability that an intervention is more cost-effective compared with the alternative intervention(s) over a range of ceiling values (λ), representing the willingness to pay (WTP) for an additional unit of effectiveness (such as $/QALY) [29, 30].

**Fig. 8:**
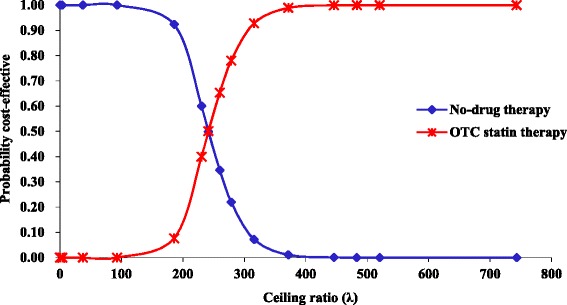
CEAC for the base-case scenario (governmental tariffs)

Figure 8 shows the CEAC for the base-case scenario, based on the QALY values for OTC statin therapy when compared to no-drug therapy.

This figure represents the traditional ‘textbook’ case of a CEAC in which OTC statin therapy is both more costly and more effective than no-drug therapy. As none of the pairs represent cost-saving the CEAC cuts the Y-axis at zero. Also as the whole density involves health gains the CEAC asymptotes to 1 [31].

A cross-over in acceptability between treatments is seen at a WTP of $240/QALY. This shows that the probability of no-drug therapy being more cost-effective than OTC statin therapy for the primary prevention of CVD is higher only if the WTP is less than this amount.

## Discussion

There is extensive literature regarding the usefulness of the use of statins for the primary prevention of CVD. Considering the increasing risk of MI in the Iranian population, for the first time, we evaluated the cost-utility and cost-effectiveness of the use of an OTC low dose statin (simvastatin 10 mg) for the primary prevention of MI, among middle aged Iranian men with an average 10-year CVD risk.

We found that for the base-case scenario, simvastatin 10 mg had a cost-utility of $1113 per additional QALY ($979 with private tariffs) and a cost-effectiveness of $935 per additional LYG ($823 with private tariffs) for the primary prevention of MI in 45-year-old men with a 15 % 10-year CVD risk.

Although the total cost for health service is higher in private sector, the difference between intervention and no-intervention groups in private sector is less than the same difference in public sector.

The performed scenario analyses produced incremental costs of no more than $1568 per additional outcome per patient.

The results of PSA and one-way sensitivity analyses showed the robustness of our results.

We did not find any other study for the cost-effectiveness of the use of OTC statins in Iran. The results of our study is consistent with a previously published modeling study estimated an incremental QALYs of 0.06 (with a discount rate of 5 %) for the use of simvastatin 10 mg for primary prevention in a male patient with 15 % 10-year CVD risk. This study differs from ours in discounting rate, considered health states and treatment pathways [32].

No threshold has been determined in Iran for the cost-effectiveness of health-related interventions. However, considering the 2013 reported GDP per capita for Iran by World Bank ($4763) [28], the estimated ICERs of this study show that the evaluated intervention can be considered highly cost-effective according to WHO recommendations [33].

This analysis has several limitations. We adopted a payer's perspective and considered only direct medical costs due to limited data on indirect costs, as well as the existence of differences among patients in their socioeconomic status and health insurance coverage. We did not model cardiovascular events other than MI and patients with cardiovascular risk factors such as hypertension or diabetes. Although we discounted the results in different scenarios, the effect of inflation was not taken into account. We also did not have male-specific data on costs. Due to the limitation of access to imported branded statins, only domestic generic statin costs were considered in the model. However, our model's use of probabilities varying with age, made it more realistic, although much more complex. We also attempted to deal with uncertainties by performing one-way and probabilistic sensitivity analyses and performing different scenarios.

As a final point, we believe that our modeling study has important implications for decision-makers in health practice and policy, particularly in Iran.
